# Protective efficacy of concomitant day-old vaccination with live-attenuated infectious bronchitis virus (IBV) vaccines (1/96 and H120 + PHY.LMV.42) against a Thai QX-like IBV challenge in commercial broilers

**DOI:** 10.14202/vetworld.2026.3256-3267

**Published:** 2026-07-28

**Authors:** Thotsapol Thomrongsuwannakij, Boonkhwan Wongyounoi, Doan Hoang Phu, Niwat Chansiripornchai

**Affiliations:** 1 Akkhraratchakumari Veterinary College, Walailak University, Nakhon Si Thammarat 80160, Thailand.; 2 Center for One Health, Walailak University, Nakhon Si Thammarat 80160, Thailand.; 3 GFPT Public Company Limited, Thailand.; 4 Faculty of Animal Science and Veterinary Medicine, Nong Lam University, Ho Chi Minh City 71309, Vietnam.; 5 Avian Health Research Unit, Department of Veterinary Medicine, Faculty of Veterinary Science, Chulalongkorn University, Bangkok 10330, Thailand.

**Keywords:** broiler chickens, cross-protection, infectious bronchitis virus, live-attenuated vaccine, QX-like strain, respiratory disease, Thailand, vaccine efficacy

## Abstract

**Background and Aim:**

Infectious bronchitis virus (IBV) remains a major cause of respiratory disease and economic loss in commercial poultry production, particularly with the emergence and widespread circulation of QX-like variants. In Thailand, these strains continue to challenge existing vaccination programs because of their genetic diversity and limited cross-protection afforded by conventional vaccines. This study evaluated the protective efficacy of concomitant day-old administration of two live-attenuated IBV vaccines (1/96 and H120 + PHY.LMV.42) against challenge with a Thai QX-like IBV strain in commercial broiler chickens.

**Materials and Methods:**

Seventy day-old Cobb500 broiler chickens were randomly assigned to vaccinated, positive control, and negative control groups. Birds in the vaccinated group received concomitant ocular administration of the 1/96 and H120 + PHY.LMV.42 vaccines at hatch and were challenged at 21 days of age with a Thai QX-like IBV strain (10⁴ median embryo infectious dose [EID₅₀]/0.1 mL). Protective efficacy was evaluated using tracheal ciliostasis protection score (CPS), binomial protection score (BPS), reverse transcription-polymerase chain reaction for viral detection, serological responses determined by enzyme-linked immunosorbent assay, and body weight measurements. Statistical analyses were performed using one-way analysis of variance or the Kruskal–Wallis test, with p < 0.05 considered statistically significant.

**Results:**

Vaccinated broilers exhibited significantly improved tracheal protection compared with unvaccinated challenged controls, achieving a CPS of 58.5% and a BPS of 100%, whereas positive control birds demonstrated complete ciliostasis (CPS = 0%). Viral detection was reduced in vaccinated birds, although the reduction was not statistically significant. Vaccinated birds also developed significantly higher antibody titers after challenge than negative controls (p < 0.05), indicating enhanced humoral immunity. Body weight at 26 days of age remained significantly lower in both challenged groups than in unchallenged controls, suggesting that vaccination only partially mitigated the adverse effects of IBV infection on growth performance. No mortality occurred during the experimental period.

**Conclusion:**

Concomitant day-old administration of the 1/96 and H120 + PHY.LMV.42 live-attenuated vaccines provided measurable cross-protection against a Thai QX-like IBV challenge by preserving tracheal ciliary function, enhancing antibody responses, and reducing viral detection in commercial broilers. This hatchery-applicable vaccination strategy represents a practical approach for improving early protection against circulating QX-like IBV strains in Thailand. Nevertheless, protection remained partial, and additional studies involving larger populations, diverse circulating variants, quantitative viral load assessment, and commercial field validation are required to optimize vaccination strategies and confirm long-term protective efficacy.

## INTRODUCTION

Infectious bronchitis (IB) is a highly contagious and economically important viral disease of poultry worldwide. It is caused by infectious bronchitis virus (IBV), a single-stranded RNA virus belonging to the genus *Gammacoronavirus* within the family *Coronaviridae* [1]. IBV primarily infects the respiratory tract but may also involve the renal and reproductive systems, resulting in substantial economic losses because of increased morbidity and mortality, impaired growth performance, reduced egg production, and poor egg quality [2, 3]. Transmission occurs through aerosolized respiratory secretions, direct contact with infected birds, and indirect exposure to contaminated feed, water, litter, equipment, and other fomites [4].

Co-infections with bacterial pathogens such as *Escherichia coli*, *Mycoplasma gallisepticum*, and *Mycoplasma synoviae* can aggravate the clinical severity of IB, increase mortality, and further complicate disease management [5]. Nephropathogenic IBV strains are of particular concern because they induce severe renal lesions that contribute to elevated mortality in broiler flocks [6]. Since its first identification in China in 2004, the QX variant of IBV has spread extensively throughout Asia, Europe, and other poultry-producing regions [7]. QX-like strains are associated with respiratory disease, nephropathogenic lesions, and reproductive disorders, particularly in young birds, resulting in long-term developmental abnormalities, including cystic oviducts and impaired reproductive performance [8–10].

In Thailand, several IBV genotypes, including TH1, TH2, and Massachusetts-type strains, have been identified [11]. The TH2 genotype is genetically closely related to the QX lineage and has been implicated in major disease outbreaks, posing a continuing challenge to effective disease control [12]. The extensive genetic diversity of IBV, driven primarily by mutations and recombination within the spike (*S1*) gene, facilitates immune evasion and reduces vaccine effectiveness [13]. Recombination among circulating IBV strains further promotes the emergence of novel serotypes that may escape immunity induced by existing vaccines [14]. Consequently, continuous molecular surveillance of circulating IBV strains is essential for developing and updating effective vaccination strategies.

Molecular epidemiological studies have demonstrated that QX-like IBV strains gradually replaced indigenous Thai IBV lineages after 2009 and have since become predominant in several regions of Thailand [10, 11]. These viruses are associated with respiratory disease, nephropathogenic lesions, impaired growth performance, and increased mortality in commercial broilers, resulting in considerable economic losses. Because both classical and variant IBV strains currently co-circulate in Thailand, vaccination strategies based on the protectotype concept have received increasing attention. This concept combines antigenically distinct vaccine strains to broaden heterologous protection against genetically diverse field viruses [15]. Accordingly, the combination of variant strain 1/96 and Massachusetts-type strain H120 has been proposed as a practical strategy to enhance tracheal protection against QX-like viruses. However, the effectiveness and potential limitations of concomitant administration of these vaccines under commercial broiler conditions remain insufficiently characterized.

Despite the widespread use of live-attenuated IBV vaccines, outbreaks caused by QX-like strains continue to occur in Thailand, indicating that currently implemented vaccination programs do not consistently provide adequate cross-protection against genetically diverse field viruses [16, 17]. Although previous investigations have evaluated single vaccine strains or sequential vaccination programs against heterologous IBV challenge, evidence regarding the simultaneous administration of two antigenically distinct live vaccines at day-old is limited, particularly in commercial broilers with maternally derived antibodies. Furthermore, most previous Thai studies have been conducted using specific-pathogen-free (SPF) chickens or vaccination schedules that differ from routine hatchery practices, thereby limiting the direct applicability of their findings to commercial production systems. Consequently, there remains a need to evaluate whether concomitant hatch-day vaccination with commercially available live-attenuated vaccines can provide effective cross-protection against currently circulating Thai QX-like IBV strains under conditions that closely resemble commercial broiler production.

Therefore, this study aimed to evaluate the protective efficacy of concomitant hatch-day vaccination with two commercially available live-attenuated IBV vaccines, Cevac IBird (strain 1/96; Ceva Animal Health, Libourne, France) and Cevac Vitabron L (strains H120 + PHY.LMV.42; Ceva Animal Health), against challenge with a Thai QX-like IBV strain in commercial broiler chickens. Protective efficacy was assessed by evaluating tracheal ciliary protection, viral detection by reverse transcription-polymerase chain reaction (RT-PCR), serological responses, clinical performance, and growth characteristics following challenge. We hypothesized that simultaneous exposure to two antigenically distinct live-attenuated vaccine strains at hatch would induce broader heterologous immunity and confer superior protection against Thai QX-like IBV infection compared with vaccination strategies based on a single vaccine strain.

## MATERIALS AND METHODS

### Ethical approval

All experimental procedures involving animals were reviewed and approved by the Chulalongkorn University Institutional Animal Care and Use Committee (IACUC Approval No. 1531069) and the Chulalongkorn University Institutional Biosafety Committee (IBC Approval No. 1531003) before commencement of the study. The research was conducted in accordance with the institutional guidelines for the ethical care and use of laboratory animals, the applicable national regulations governing animal experimentation in Thailand, and internationally accepted principles for the ethical use of animals in scientific research.

The approved protocol covered all experimental procedures, including procurement and housing of broiler chickens, administration of live-attenuated IBV vaccines, experimental challenge with the Thai QX-like IBV strain, clinical monitoring, blood and tracheal sample collection, laboratory investigations involving infectious materials, and humane euthanasia. All procedures involving the challenge virus were performed under the approved institutional biosafety regulations to ensure the safety of personnel, animals, and the surrounding environment.

Throughout the study, birds were housed in separate biosecure isolation rooms equipped with independent ventilation systems to prevent cross-contamination among experimental groups. Standard husbandry practices were maintained, and birds had unrestricted access to commercial feed and clean drinking water. Animal health and welfare were monitored at least twice daily by trained personnel, and birds exhibiting signs of severe distress, pain, or illness beyond the expected effects of the experimental infection would have received immediate veterinary attention and been humanely euthanized according to the approved humane endpoint criteria.

Vaccination, viral challenge, blood collection, and tissue sampling were performed by experienced veterinarians or trained research personnel using standardized procedures designed to minimize handling stress and discomfort. At the completion of the challenge experiment (5 days post-challenge [dpc]), all birds were humanely euthanized by carbon dioxide (CO₂) asphyxiation in accordance with the approved IACUC protocol before postmortem tissue collection. Every effort was made throughout the study to minimize animal suffering, reduce the number of animals used, and adhere to the principles of Replacement, Reduction, and Refinement (3Rs).

### Study period and location

The study was conducted from March to September 2021. The animal challenge experiment was performed at the experimental animal facility of the Faculty of Veterinary Science, Chulalongkorn University, Nakhon Pathom Campus, Thailand. Laboratory analyses, including virological and serological assays, were carried out at the Faculty of Veterinary Science, Chulalongkorn University, Bangkok, Thailand.

### Study design

A randomized controlled challenge study was performed to evaluate the protective efficacy of concomitant hatch-day vaccination with two live-attenuated IBV vaccines against a Thai QX-like IBV strain. Seventy commercial day-old Cobb500 broiler chickens were randomly allocated to vaccinated, positive control, and negative control groups. Birds in the vaccinated group received concomitant ocular vaccination on the day of hatch, whereas birds in the control groups remained unvaccinated. At 21 days of age, vaccinated and positive control birds were challenged with a Thai QX-like IBV strain, while the negative control birds remained unchallenged. Protective efficacy was assessed by evaluating tracheal ciliostasis, viral detection by RT-PCR, serological responses, and body weight.

### Viruses and vaccines

**Challenge virus:** The challenge virus was IBV strain THA80151 (GenBank accession number FJ156075) [11]. The virus was propagated in 9- to 11-day-old SPF embryonated chicken eggs and used at passage 4 in the challenge experiment. Following incubation, allantoic fluids were harvested from embryos exhibiting characteristic IBV lesions and clarified by centrifugation. Virus stocks were aliquoted and stored at −80°C until use. Viral infectivity was determined by endpoint titration in SPF embryonated chicken eggs using the Reed and Muench method and expressed as the median embryo infectious dose (EID₅₀)/mL. Based on the calculated titer, the virus stock was diluted in sterile phosphate-buffered saline (pH 7.2) to obtain a final challenge dose of 10⁴ EID₅₀ per 0.1 mL.

**Vaccines: Two commercial live-attenuated vaccines were used in this study:** Cevac IBird (strain 1/96; Batch No. 0604D4U2; Ceva Animal Health, Libourne, France) at a dose of 0.03 mL (10³^.^⁸ EID₅₀/bird) and Cevac Vitabron L (strains H120 + PHY.LMV.42; Batch No. 0603D3SKF; Ceva Animal Health, Libourne, France) at a dose of 0.03 mL (10⁰^.^³ EID₅₀/bird for IBV and 10³^.^⁰ EID₅₀/bird for Newcastle disease virus). Vaccines were transported and stored under the manufacturer's recommended cold-chain conditions until reconstitution with sterile diluent immediately before administration. All vaccine doses were administered within 1 h after reconstitution to preserve vaccine viability.

### Chickens and experimental design

Seventy commercial day-old Cobb500 broiler chickens, which more closely represent commercial production systems than SPF chickens, were obtained from a commercial hatchery (GFPT Public Company Limited, Samut Prakan, Thailand) and transported to the experimental animal facility at Chulalongkorn University, Nakhon Pathom Campus.

Birds were randomly assigned to three experimental groups and housed separately in accordance with the experimental design (Table 1). The mean maternally derived IBV antibody titer at 1 day of age was 2,565.5 ± 712.55 enzyme-linked immunosorbent assay units (BioChek B.V., Reeuwijk, the Netherlands).

On the day of hatch, birds in Group A received concomitant ocular vaccination with Cevac IBird (strain 1/96) and Cevac Vitabron L (strains H120 + PHY.LMV.42) according to the manufacturer's recommendations. Birds in Groups B and C remained unvaccinated. This concomitant vaccination strategy differs from most previous studies, which evaluated either single vaccine strains or sequential vaccination schedules.

At 21 days of age, birds in Groups A and B were challenged with the Thai QX-like IBV strain by the intraocular and intranasal routes, whereas birds in Group C served as the unchallenged negative control. At 5 dpc, all birds were humanely euthanized using CO₂ asphyxiation in accordance with the approved IACUC protocol. Tracheal tissues were collected for assessment of ciliary activity and RT-PCR analysis.

**Table 1:** Experimental design.

**Vaccination at 1 day of age**	**Group**	**Number of ** **broilers**	**Challenge ** **age ** **(** **days** **)**	**Birds for ** **ciliostasis** **(** **5 ** **dpc** **)**	**Birds for ** **RT** **-** **PCR** **(** **5 ** **dpc** **)**
Cevac IBird (1/96) + Cevac Vitabron L (H120 + PHY.LMV.42)	A	20	21	10	10
None	B	20	21	10	10
None	C	20	NA	10	10

dpc = Days post-challenge; NA = Not applicable; RT-PCR = Reverse transcription-polymerase chain reaction.

### Housing of the animals

Each experimental group was housed in an independent isolation room within the Livestock Hospital, Faculty of Veterinary Science, Chulalongkorn University, Nakhon Pathom, Thailand. Each room had an independent ventilation system to prevent cross-contamination among treatment groups.

Strict biosecurity procedures were maintained throughout the study. Personnel followed a one-way movement protocol from non-challenged to challenged rooms and changed dedicated protective clothing, gloves, boots, and disposable equipment before entering each room.

Throughout the experiment, birds had unrestricted access to commercial feed (Betagro Public Company Limited, Bangkok, Thailand) and clean drinking water.

### Vaccination

Cevac IBird and Cevac Vitabron L were reconstituted in chilled, sterile demineralized water to obtain a single commercial dose per bird. The reconstituted vaccines were maintained at 3-5°C until administration. Vaccines were administered by the ocular route, with each bird receiving two drops (0.03 mL/drop), equivalent to one commercial dose.

### IBV challenge

Frozen allantoic fluid containing the Thai QX-like IBV strain was thawed and diluted in chilled, sterile, demineralized water to a final concentration of 10⁴ EID₅₀/0.1 mL. The virus suspension was maintained under refrigerated conditions until use. Each bird received two ocular drops (0.05 mL/drop), providing a total challenge volume of 0.1 mL.

### Ciliary activity test

The ciliary activity test was performed as previously described by *Cook et al.* [19] with minor modifications. Each trachea was divided into proximal, middle, and distal regions and sectioned into five rings (two proximal, one middle, and two distal), each measuring <2 mm in thickness. Individual rings were placed into separate wells of a 24-well culture plate containing minimum essential medium/Earle's balanced salt solution medium (Merck KGaA, Darmstadt, Germany).

Ciliary activity was evaluated microscopically by an investigator blinded to treatment allocation. Each ring was scored using a 0-4 scale:

1. 0 = all cilia beating

2. 1 = approximately 75% ciliary activity

3. 2 = approximately 50% ciliary activity

4. 3 = approximately 25% ciliary activity

5. 4 = complete ciliostasis

The maximum ciliostasis score for each trachea was 20 (4 × 5 rings).

Birds were considered protected when the total ciliostasis score was <10.

The ciliostasis protection score (CPS) was calculated as:


\[
CPS=100-\frac{100\times \sum _{}^{}{individual ciliostasis scores}}{n\times 20}
\]

where *n* represents the number of birds examined.

The binomial protection score (BPS) was calculated as the percentage of birds exhibiting >50% ciliary activity (ciliostasis scores of 0-2), as described by *de Wit et al.* [20].

### IBV detection by RT-PCR

At 5 dpc, tracheal samples were collected from 10 randomly selected birds in each group for RT-PCR detection of IBV.

Samples were homogenized in 10% PBS and centrifuged at 1,800 × g for 10 min. Viral RNA was extracted from the clarified supernatant using the HiYield™ Viral Nucleic Acid Extraction Kit (RBC Bioscience Corp., New Taipei City, Taiwan).

Primers previously described by Sarueng *et al.* [15] were used to differentiate the challenge strain (QX-like THA80151) from the vaccine strains (H120 and 793/B). Primer sequences were:

1. **F1547:** 5′-TAATGAAACTGGTTCTCAGCC-3′

2. **R1691:** 5′-GCGGTACTATTTGCTTAATAA-3′

One-step RT-PCR consisted of reverse transcription at 48°C for 45 min, initial denaturation at 94°C for 5 min, followed by 35 amplification cycles comprising denaturation at 94°C for 30 s, annealing at 56°C for 30 s, and extension at 72°C for 1 min, with a final extension at 72°C for 10 min [15].

Amplified products were separated on 1.2% agarose gels stained with ethidium bromide (0.5 μg/mL) and visualized using an ultraviolet transilluminator.

### Serological evaluation

Blood samples were collected at 1, 7, 14, 21, and 26 days of age. Serum samples were separated and analyzed for IBV-specific antibodies using a commercial enzyme-linked immunosorbent assay kit (BioChek B.V., Reeuwijk, the Netherlands) to evaluate humoral immune responses following vaccination and challenge.

### Statistical analysis

Continuous data were evaluated for normality using the Shapiro-Wilk test and visual inspection of Q-Q plots. Body weight and antibody titers were analyzed using one-way analysis of variance, followed by Duncan's multiple range test for pairwise comparisons. Ciliostasis scores and RT-PCR detection data were analyzed using the Kruskal–Wallis test.

All statistical analyses were performed using IBM SPSS Statistics version 29 (IBM Corp., Armonk, NY, USA). Statistical significance was established at p < 0.05.

## RESULTS

### Body weight

No significant differences in body weight were observed among the experimental groups at 21 days of age, indicating that vaccination did not adversely affect early growth performance (Table 2). However, at 26 days of age (5 dpc), birds in the negative control group exhibited significantly greater body weights than those in the vaccinated and positive control groups (p < 0.05), whereas no significant difference was detected between the vaccinated and positive control groups (Table 2). These findings indicate that challenge with the Thai QX-like IBV strain adversely affected growth performance regardless of vaccination status, although vaccination provided protection against other disease-associated parameters. No mortality was observed throughout the experimental period.

### Ciliostasis and CPS

At 26 days of age (5 dpc), tracheal samples were collected for ciliostasis assessment and RT-PCR analysis. Birds in the positive control group exhibited significantly higher ciliostasis scores than those in the vaccinated and negative control groups (p < 0.05) (Table 2). Notably, concomitant hatch-day vaccination achieved a CPS of 58.5%, whereas the unvaccinated challenged group exhibited complete ciliostasis (CPS = 0%), demonstrating markedly superior preservation of tracheal ciliary function following vaccination.

The vaccinated group exhibited a substantially higher CPS (58.5%) than the positive control group (0%), indicating significant tracheal protection following heterologous challenge. Furthermore, the BPS was identical in the vaccinated and negative control groups (100%), indicating comparable preservation of ciliary activity in protected birds.

### Viral detection

RT-PCR analysis demonstrated that birds in the positive control group had the highest viral detection rate, which was significantly greater than that observed in the negative control group (p < 0.05) (Table 2). Although the vaccinated group showed a lower viral detection rate than the positive control group, the difference was not statistically significant. These findings suggest that concomitant vaccination partially suppressed viral replication but did not completely prevent infection following challenge with the Thai QX-like IBV strain.

**Table 2:** Average body weight, ciliostasis scores, and RT-PCR detection at 26 days of age.

**Group**	**Body weight ** **(** **g** **) ** **at 21 days ** **(** **mean ± SD; n ** **= ** **20** **/** **group** **)**	**Body weight ** **(** **g** **) ** **at 26 days ** **(** **mean ± SD; n ** **= ** **20** **/** **group** **)**	**Average ** **ciliostasis** ** score** **/** **bird ** **(** **n ** **= ** **10** **)**	**CPS**	**BPS**	**RT** **-** **PCR detection ** **(** **n ** **= ** **10** **)**
Vaccinated	933.75 ± 97.82ᵃ	1,109.50 ± 113.63ᵃ	8.3ᵃ	58.5	100	7ᵃ
Positive control	932.75 ± 100.87ᵃ	1,200.75 ± 64.30ᵃ	20ᵇ	0	0	10ᵃ
Negative control	984.75 ± 73.10ᵃ	1,230.50 ± 83.02ᵇ	0ᶜ	100	100	0ᵇ

Values are presented as mean ± SD where applicable. Different superscript letters (ᵃ–ᶜ) within the same column indicate significant differences (p < 0.05). BPS = Binomial protection score; CPS = Ciliostasis protection score; RT-PCR = Reverse transcription-polymerase chain reaction; SD = Standard deviation.

### Serological evaluation

**Figure 1:**
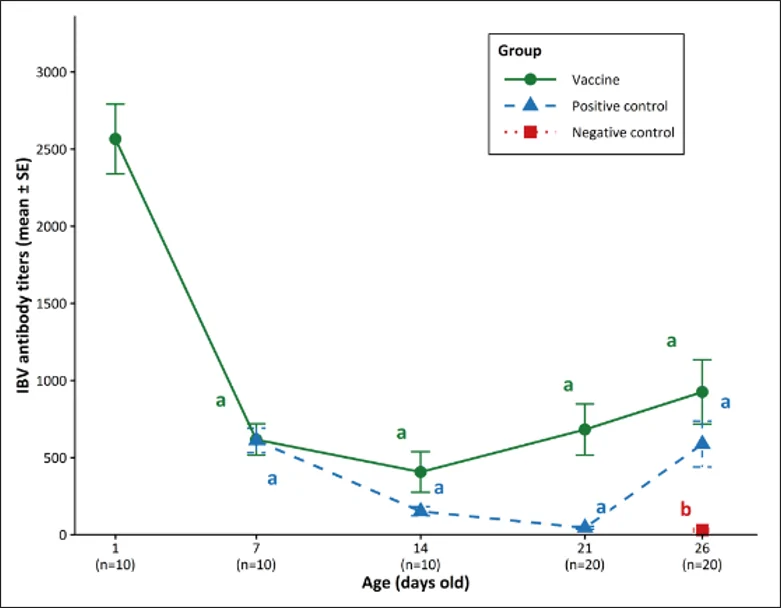
Kinetics of serum antibody responses against IBV in broiler chickens vaccinated at hatch with concomitant administration of two live-attenuated IBV vaccines (Cevac IBird and Cevac Vitabron L). Antibody titers were measured by enzyme-linked immunosorbent assay at 1, 7, 14, 21, and 26 days of age.

Maternal antibody titers were detected in all experimental groups at 1 day of age (Figure 1). Antibody titers gradually declined in all groups during the first 3 weeks of life as maternally derived antibodies waned. Although the vaccinated group maintained numerically higher antibody titers than the positive and negative control groups at 14 and 21 days of age, these differences were not statistically significant.

At 26 days of age (5 dpc), both the vaccinated and positive control groups exhibited significantly higher antibody titers than the negative control group (p < 0.05), indicating antibody responses induced by vaccination and/or challenge infection. The vaccinated group consistently exhibited the highest antibody titers following challenge, suggesting enhanced humoral immunity after concomitant hatch-day vaccination.

## DISCUSSION

### Overall protective efficacy of concomitant vaccination

To our knowledge, this is the first study to demonstrate protective efficacy against a Thai QX-like IBV strain following concomitant hatch-day administration of two commercially available live-attenuated IBV vaccines in commercial broilers. This vaccination strategy addresses the practical limitations associated with sequential vaccination programs in modern hatcheries while providing early protection during a critical period of susceptibility.

The present study evaluated the protective efficacy of concomitant administration of Cevac IBird (strain 1/96) and Cevac Vitabron L (strains H120 + PHY.LMV.42) in broilers challenged with a Thai QX-like IBV strain at 21 days of age. The results demonstrated that this vaccination strategy provided measurable protection against heterologous challenge, particularly by preserving tracheal ciliary function, enhancing humoral immune responses, and partially reducing viral detection.

### Tracheal protection and preservation of ciliary function

One of the principal findings of this study was that CPS was significantly higher in vaccinated birds than in the positive control group. Vaccinated birds achieved a CPS of 58.5%, whereas the unvaccinated challenged birds exhibited complete ciliostasis (CPS = 0%), indicating severe tracheal damage. The mucociliary apparatus constitutes one of the primary defense mechanisms of the avian respiratory tract against inhaled pathogens, including IBV [21, 22]. Preservation of ciliary activity facilitates mucociliary clearance, thereby limiting viral persistence and reducing tissue damage.

The markedly higher CPS observed in vaccinated birds indicates that concomitant vaccination effectively preserved tracheal integrity following heterologous challenge. In contrast, the complete loss of ciliary activity in unvaccinated challenged birds highlights the susceptibility of unprotected birds to severe respiratory injury following infection with Thai QX-like IBV.

Similarly, vaccinated birds achieved a BPS of 100%, whereas the positive control group showed no protected birds. These findings indicate that concomitant vaccination maintained functional tracheal cilia in a substantially greater proportion of birds, which may reduce disease severity and limit within-flock virus transmission [23].

### Viral detection following challenge

RT-PCR analysis demonstrated a lower viral detection rate in the vaccinated group than in the positive control group, although the difference was not statistically significant. These findings suggest that concomitant vaccination did not completely prevent infection but partially suppressed viral replication following heterologous challenge.

Previous studies have similarly demonstrated that heterologous vaccination strategies may reduce viral replication and disease severity without completely preventing infection [9, 18]. Partial suppression of viral replication may nevertheless reduce environmental virus contamination, improve flock health, and lessen production losses under commercial conditions. However, because RT-PCR was used for qualitative detection, the present findings should be interpreted as reduced viral detection rather than a confirmed reduction in viral load or shedding.

### Humoral immune response

The serological findings further supported the immunogenic effect of concomitant vaccination. Vaccinated birds developed significantly higher antibody titers than the negative control group following challenge, indicating effective stimulation of humoral immunity. Although maternally derived antibody titers declined with age across all groups, vaccinated birds maintained numerically higher antibody responses than control birds at 14 and 21 days of age.

The significantly higher antibody titers in vaccinated birds at 5 dpc suggest that vaccination primed the immune system for a rapid anamnestic response following challenge. However, the positive control group also developed significantly higher titers than the negative control group at this time point, indicating that challenge infection contributed to the observed serological response. Therefore, antibody titers alone cannot fully distinguish vaccine-induced protection from the immune response to infection.

### Growth performance following challenge

No significant differences in body weight were detected among the groups before challenge, indicating that hatch-day vaccination did not adversely affect early growth performance. However, at 5 dpc, birds in both the vaccinated and positive control groups had significantly lower body weights than those in the negative control group.

These findings indicate that the Thai QX-like IBV challenge adversely affected short-term growth performance regardless of vaccination status. The absence of a significant difference between the vaccinated and positive control groups suggests that vaccination did not prevent challenge-associated growth depression during the short observation period. Therefore, although vaccination preserved tracheal ciliary activity and enhanced antibody responses, its benefit was not reflected in improved body weight by 5 dpc.

### Comparison with previous studies and possible protective mechanisms

The emergence of genetically diverse IBV variants continues to complicate disease control. The extensive genetic variability of IBV, driven by mutation and recombination, may reduce vaccine-induced cross-protection and necessitates continuous evaluation of vaccination programs.

The combination of Cevac IBird and Cevac Vitabron L represents a practical application of the protectotype concept, in which antigenically distinct vaccine strains are combined to broaden heterologous protection [15]. The present findings are consistent with those reported by Sarueng *et al.* [15] and Thomrongsuwannakij *et al.* [24], who demonstrated improved heterologous protection following administration of two IBV vaccine strains. Whereas those studies evaluated vaccines administered at different ages, the present investigation demonstrates that concomitant hatch-day administration can also provide measurable protection in commercial broilers.

The previous studies were conducted in commercial broilers and SPF chickens, respectively, suggesting that the benefits of dual-strain vaccination may extend across different experimental and production settings. Simultaneous priming with Massachusetts-type H120 and 1/96 (793/B) strains may broaden heterologous immunity by stimulating responses to a wider range of IBV antigens, consistent with the protectotype concept described by Cook *et al.* [19] and further supported by de Wit *et al.* [20].

Exposure to antigenically distinct vaccine strains may increase immune recognition of conserved IBV epitopes and promote cross-reactive CD4⁺ and CD8⁺ T-cell responses against relatively conserved viral proteins, including the nucleocapsid and membrane proteins [25, 26]. Replication of live vaccine viruses in the respiratory tract may also induce local mucosal immunity, including secretory immunoglobulin A production, which can interfere with viral attachment and replication at mucosal surfaces [27–29]. In addition, local memory T-cell responses generated after vaccination may facilitate more rapid viral clearance following heterologous challenge [29, 30]. Collectively, these mechanisms may complement systemic humoral immunity and contribute to the protection observed after combined Massachusetts and 793/B vaccination programs [19, 21, 31].

### Vaccine strain selection and IBV evolution

Recent studies evaluating live-attenuated IBV vaccines further emphasize the importance of vaccine strain selection in optimizing protection against emerging variants. Kilany *et al.* [32] demonstrated that a live-attenuated GI-23 vaccine was safe, did not revert to virulence, and provided effective protection against homologous challenge. Although the genotype-matched strategy evaluated by Kilany *et al.* [32] differs from the protectotype-based strategy used in the present study, both approaches highlight the importance of antigenic compatibility between vaccine strains and circulating field viruses.

Elfeil *et al.* [33] reported differences in protection among commercially available vaccination programs against QX challenge, indicating that vaccine strain selection and vaccination strategy can substantially influence heterologous protection. Similarly, Shosha *et al.* [34] demonstrated extensive genetic diversity and recombination among circulating IBV strains, particularly within the GI-19 and GI-23 lineages. These findings reinforce the need for continuous molecular surveillance and periodic reassessment of vaccination programs.

Emerging evidence also indicates that recombination involving vaccine-related lineages may contribute to the evolution of novel IBV variants. Huang *et al.* [35] identified a naturally occurring recombinant virus containing genomic material derived from a 4/91-like vaccine lineage, which showed greater virulence than its parental field strain. These observations emphasize the need to balance the benefits of live-attenuated vaccines against the ongoing monitoring of vaccine-derived and field virus evolution.

### Concurrent IBV and Newcastle disease virus (NDV) vaccination

In the present study, concomitant administration of live IBV and NDV vaccine components at 1 day of age was associated with improved protection against subsequent challenge with a Thai QX-like IBV strain. These findings suggest that hatchery vaccination may provide a practical foundation for early protection during the period when broilers are highly susceptible to respiratory infection.

Nevertheless, potential interference between live IBV and NDV vaccines administered concurrently should be considered. The magnitude of vaccine interaction may depend on vaccine strain, route of administration, maternally derived antibody levels, and replication kinetics. Competition for respiratory epithelial cells and the induction of innate antiviral responses could potentially alter the replication of one or both vaccine viruses.

However, concurrent administration does not necessarily compromise protective immunity. Ball *et al.* [36] demonstrated that day-old broilers receiving NDV VG/GA-Avinew concurrently with IBV H120 and CR88 developed protective NDV antibody responses while maintaining high levels of ciliary protection against both M41 and QX challenge strains. Collectively, these findings suggest that concurrent administration of appropriately selected IBV and NDV vaccine strains can provide satisfactory protection, although the interactions between vaccine viruses require further evaluation under commercial conditions.

### Practical implications for commercial poultry production

The present findings have important implications for commercial poultry production in Thailand, where QX-like IBV strains continue to circulate. Because IBV infection commonly occurs during the first weeks of life, establishing early protective immunity is critical for reducing respiratory disease, secondary bacterial infections, impaired growth, and production losses.

Hatchery-based concomitant vaccination offers several operational advantages over farm-level administration, including standardized vaccine delivery, improved dose uniformity, reduced labor requirements, lower handling stress, and a reduced risk of administration errors. These advantages are particularly relevant to large integrated poultry operations in which large numbers of chicks must be vaccinated efficiently.

The protective outcomes observed in this study support the potential inclusion of the evaluated vaccine combination in an integrated IBV control program. However, vaccine selection should be guided by the genetic and antigenic characteristics of locally circulating field strains. Continuous surveillance of IBV genotypes in Thailand remains essential to determine whether existing vaccination programs provide adequate cross-protection against emerging variants.

Successful field implementation also depends on hatchery management practices, including vaccine storage and handling, reconstitution procedures, equipment calibration, dose delivery, chick quality, and biosecurity. Routine monitoring of vaccine uptake, serological responses, respiratory health, and flock performance should therefore be incorporated into commercial vaccination programs to optimize protection and minimize the economic impact of IB.

### Study strengths, limitations, and future perspectives

A major strength of this study was the use of commercial broilers with maternally derived antibodies, which provides greater practical relevance than studies conducted exclusively in SPF chickens. The concomitant hatch-day vaccination schedule also closely reflects the operational requirements of modern commercial hatcheries. In addition, protection was evaluated using complementary outcomes, including body weight, ciliostasis, CPS, BPS, RT-PCR detection, and serological responses.

Several limitations should nevertheless be acknowledged. First, the relatively small sample size may limit the generalizability of the findings to commercial field conditions. Second, the short experimental period restricted assessment to the early post-challenge phase and did not permit evaluation of long-term protection, prolonged virus persistence, later clinical progression, or sustained production performance. Third, only one Thai QX-like challenge strain was evaluated; therefore, the findings may not represent protection against other circulating genotypes or emerging recombinant variants. Fourth, RT-PCR detection was qualitative, and quantitative viral load and shedding kinetics were not determined. Finally, production performance was assessed mainly through body weight, without evaluating feed intake, feed conversion ratio, carcass characteristics, or economic outcomes.

Future studies should include larger controlled and commercial field trials, quantitative assessment of viral load and shedding duration, and challenges with multiple circulating Thai IBV genotypes. Longer follow-up periods are also required to determine the durability of protection and its effects on production performance. Comparative studies of concomitant, sequential, genotype-matched, and protectotype-based vaccination programs would further clarify the most effective strategies for controlling QX-like IBV under commercial conditions.

Although this study was performed under controlled experimental conditions, its findings may also be relevant to other regions where QX-like or related IBV variants circulate, including parts of the Middle East, Europe, and Latin America. Field validation will be essential to determine whether the observed tracheal protection and partial reduction in viral detection translate into meaningful improvements in flock health and economic performance.

## CONCLUSION

Concomitant hatch-day administration of Cevac IBird and Cevac Vitabron L provided meaningful protection against challenge with a Thai QX-like IBV strain in commercial broilers. Vaccinated birds achieved a CPS of 58.5% and a BPS of 100%, whereas the unvaccinated challenged group showed complete ciliostasis, with a CPS and BPS of 0%. Vaccination also reduced the proportion of RT-PCR-positive birds from 10/10 in the positive control group to 7/10, although this difference was not statistically significant. In addition, vaccinated birds developed higher antibody titers after challenge, confirming effective immune priming. However, vaccination did not prevent the short-term reduction in body weight associated with challenge, as both challenged groups had lower body weights than the negative control group at 5 dpc.

These findings demonstrate that concomitant administration of antigenically distinct live vaccines can preserve tracheal ciliary function and enhance immune responses against heterologous Thai QX-like IBV challenge. The strategy is operationally suitable for hatchery use and may provide a practical alternative to sequential vaccination programs in commercial broiler production. Nevertheless, the partial viral detection and absence of protection against early growth depression indicate that the vaccine combination did not provide complete protection. Larger field trials with longer follow-up periods, quantitative assessment of viral load and shedding, and evaluation against multiple circulating IBV genotypes are required. Overall, concomitant hatch-day vaccination represents a promising component of integrated IBV control programs, provided that vaccine selection is guided by continuous surveillance of locally circulating strains.

## DATA AVAILABILITY

The data generated during the study are included in the manuscript.

## GENERATIVE AI DECLARATION

The authors declare that generative artificial intelligence (AI) tools were used solely to improve language, grammar, and readability during manuscript preparation. All scientific content, data analysis, interpretation of results, and conclusions were developed and verified by the authors. The authors take full responsibility for the accuracy, integrity, and originality of the work presented, and no AI tool was listed as an author.

## AUTHORS’ CONTRIBUTIONS

TT: Conceptualization, investigation, methodology, and writing—original draft. BW: Investigation, methodology, and validation. DHP: Investigation, methodology, and data curation. NC: Conceptualization, investigation, methodology, and writing—review and editing. All authors have read and approved the final manuscript.
